# The Development of an *ex vivo* Flow System to Assess Acute Arterial Drug Retention of Cardiovascular Intravascular Devices

**DOI:** 10.3389/fmedt.2021.675188

**Published:** 2021-06-10

**Authors:** Kathryn Cooper, Claire V. Cawthon, Emily Goel, Marzieh Atigh, Uwe Christians, Saami K. Yazdani

**Affiliations:** ^1^Mechanical Engineering Department, University of South Alabama, Mobile, AL, United States; ^2^iC42 Clinical Research and Development, University of Colorado, Aurora, CO, United States; ^3^Department of Engineering, Wake Forest University, Winston-Salem, NC, United States

**Keywords:** *ex vivo*, methods, pharmacokinetics, interventional devices, drug delivery, paclitaxel, drug coated balloon

## Abstract

**Purpose:** The goal of this study was to develop an *ex vivo* system capable of rapidly evaluating arterial drug levels in living, isolated porcine carotid arteries.

**Methods:** A vascular bioreactor system was developed that housed a native porcine carotid artery under physiological flow conditions. The *ex vivo* bioreactor system was designed to quantify the acute drug transfer of catheter-based drug delivery devices into explanted carotid arteries. To evaluate our *ex vivo* system, a paclitaxel-coated balloon and a perfusion catheter device delivering liquid paclitaxel were utilized. At 1-h post-drug delivery, arteries were removed, and paclitaxel drug levels measured using liquid chromatography-tandem mass spectrometry (LC-MS/MS). Parallel experiments were performed in a pig model to validate *ex vivo* measurements.

**Results:** LC-MS/MS analysis demonstrated arterial paclitaxel levels of the drug-coated balloon-treated arteries to be 48.49 ± 24.09 ng/mg and the perfusion catheter-treated arteries to be 25.42 ± 9.74 ng/mg at 1 h in the *ex vivo* system. Similar results were measured *in vivo*, as arterial paclitaxel concentrations were measured at 59.23 ± 41.27 ng/mg for the drug-coated balloon-treated arteries and 23.43 ± 20.23 ng/mg for the perfusion catheter-treated arteries. Overall, no significant differences were observed between paclitaxel measurements of arteries treated *ex vivo* vs. *in vivo*.

**Conclusion:** This system represents the first validated *ex vivo* pulsatile system to determine pharmacokinetics in a native blood vessel. This work provides proof-of-concept of a quick, inexpensive, preclinical tool to study acute drug tissue concentration kinetics of drug-releasing interventional vascular devices.

## Introduction

Patients and primary care physicians have acknowledged the clinical burden of peripheral artery disease (PAD) and coronary artery disease (CAD) for over many decades (1, 2). Both PAD and CAD are caused by atherosclerosis, a buildup of cholesterol plaques in the inner vessel wall (3). Due to atherosclerosis affecting multiple vascular beds, different therapeutic approaches are essential. Percutaneous intervention (balloon angioplasty and stenting) has been the standard treatment for this arterial disease for the past 25 years. The use of drug-eluting stents (DES) was a major breakthrough in reducing the risk of restenosis following stent-induced injury (4, 5). This injury induces the inflammatory response that in turn activates the restenotic cascade; amidst this cascade, neointimal proliferation, extracellular matrix production, and reendothelization occur (6, 7). To combat the unwanted growth, DES utilize the stent scaffold to deliver antiproliferative drugs over several weeks to months (8).

In coronary artery application, clinical results of second-generation DES show the risk of restenosis to be <10%, yielding the DES five times more effective than the uncoated bare-metal stent (9, 10). This success is credited to the metallic scaffold that holds open the arterial lumen and the drugs that reduce the smooth muscle cell proliferation that leads to restenosis. Conversely, DES have failed to have the same clinical success for the treatment of PAD (11). This shortfall is attributed to the stents' high risk of strut fracture, mediated by the biomechanical stress (flexion and extension) that peripheral arteries undergo in the lower extremities (12, 13).

Stent fractures in combination with poor stent deployment alter drug release kinetics. Drugs eluted from stents are also constrained by the strut-to-artery surface ratio and thus are only able to deliver antiproliferative drugs to <20% of the luminal surface (14). Furthermore, small diameter peripheral vessels, often occluded in diabetic patients, are not conducive to stent intervention which precludes their use in below-the-knee applications. Revascularization of these arteries are essential for limb salvage in diabetic patients (15, 16). All together, these limitations of DES have revived ideas of delivering antiproliferative drugs without the use of a metallic stent platform in the treatment of PAD.

Alternatives for DES include drug-coated balloons (DCBs), perfusion catheters and other drug-delivery platforms (17–19). These devices are adept at treating various vascular beds including those affected in PAD (20–23). The fundamental approach of these non-stent platforms is to deliver antiproliferative drugs, typically paclitaxel, directly to the lesion site. Regardless of the approach, the success of non-stent drug delivery devices directly correlates to the retention of paclitaxel at the lesion post-procedure. Several studies have been published showing a variation of parameters to optimize and increase arterial drug retention. These variables include the use of drug carriers (excipients), duration of delivery, balloon pre-treatments, and balloon coating techniques (24–28).

Preclinical evaluation of drug-delivery devices is vital to understanding the safety and efficacy of these devices. Currently, biological testing of these devices is mostly limited to *in vivo* animal models. While these procedures are effective, the evaluation of non-stent drug delivery systems is lacking an *ex vivo* system capable of testing pharmacokinetic performance in a biologically relevant model. The success of DCBs and perfusion catheters is dependent on the initial acute transfer and the temporal retention of the therapeutic drug into the vessel, underscoring the need for pharmacokinetic evaluation in these non-stent delivery systems.

The use of organ culture of vascular tissue to evaluate stent performance is well-established as there are currently no man-made vessels that can duplicate the cellular organization, structure, and elasticity of the native artery. Swanson et al., were one of the first groups to use an *ex vivo* organ culture model to study the integration of the stent and the host artery in living tissue (29). They evaluated drug uptake and cellular proliferation in explanted human internal mammary artery. Characterization of stent-induced vascular injury response using an *ex vivo* arterial perfusion model has also been performed using harvested porcine carotid arteries (30, 31). Harvested porcine arteries can maintain functionality up to 7 days under perfusion conditions (31). More recently, explanted rabbit carotid arteries have been used to evaluate the degradation and cellular response to biodegradable stents (32).

This study was designed to evaluate non-stent drug delivery platforms, including a commercially available DCB and a perfusion catheter, *via* a novel *ex vivo* system that simulates and maintains cardiovascular conditions. Parallel *in vivo* studies were then performed using a pig ilio-femoral injury model. The data obtained from this study will establish the efficacy of the *ex vivo* bioreactor system.

## Materials and Methods

### Vessel Harvest

Porcine carotid arteries were harvested from large pigs (110–160 kg) at a local abattoir and transferred in sterile PBS with 1% antibiotic-antimitotic (Gibco, Grand Island, NY, 14072). Vessels were then rinsed in sterile PBS in a culture hood. The excess fat, connective tissue, and fascia were dissected from each vessel. Vessels were cut into ~8 cm segments and stored in 15-mL centrifuge tubes at −20°C until needed. Frozen vessels were thawed in a 37°C water bath and placed into the bioreactor system to be studied.

### *Ex vivo* Bioreactor System

**Figure 1:**
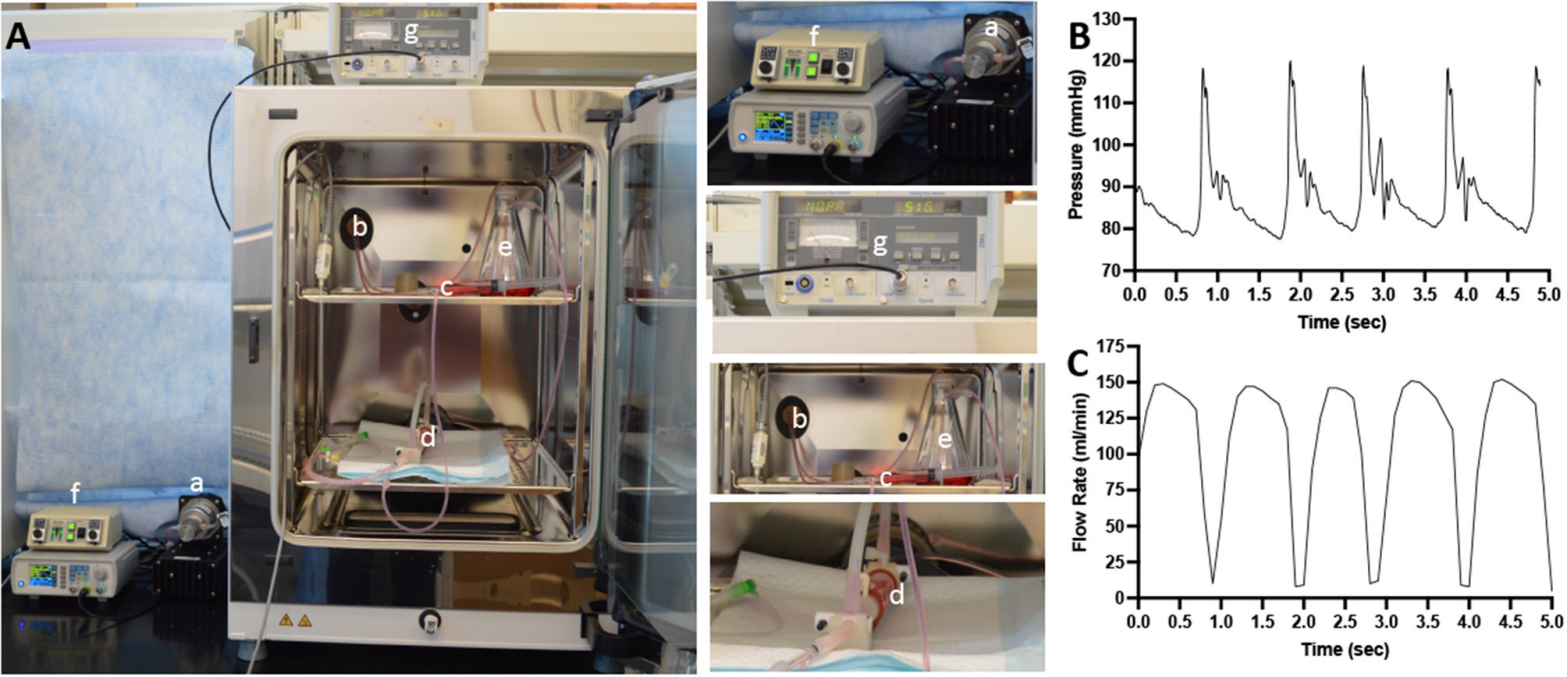
Schematic diagram of the *ex vivo* bioreactor system. **(A)** A computer controls a gear pump (a) that is capable to generate pulsatile flow conditions. The tubing from the gear pump passes through a port (b) of the CO_2_ incubator. The culture medium then passed through a low-pass filter (c) and through the lumen of the explanted arteries in the bioreactor housing compartment (d) and into the flow reservoir (e). The flow is monitored *via* pressure sensor (f) and an ultrasonic flowmeter (g). **(B)** A representative signal of the pressure within the flow system. **(C)** A representative signal of the flow rate within the flow system.

The bioreactor system used in this study consisted of a flow reservoir, gear pump (Ismatec Cole Parmer, Vernon Hills, IL), vessel housing compartment, and a distal flow constrictor. The setup is shown in Figure 1A. The pressure was monitored *via* a catheter pressure transducer (Millar Instruments, Houston, TX). The flow was monitored by an ultrasonic flow meter (Transonic Systems Inc., Ithica, NY) positioned prior to the bioreactor. A custom LabVIEW program allowed us to generate any flow waveform and control and monitor the mechanical conditions (flow and pressure) within our bioreactor system. The bioreactor medium consisted of Dulbecco's Modified Eagle's Medium (DMEM) containing low glucose, L-glutamine, 110 mg/L sodium pyruvate, pyridoxine hydrochloride 10% fetal bovine serum (Gibco), and 1% antibiotic-antimycotic (Gibco).

### Drug Delivery

**Figure 2:**
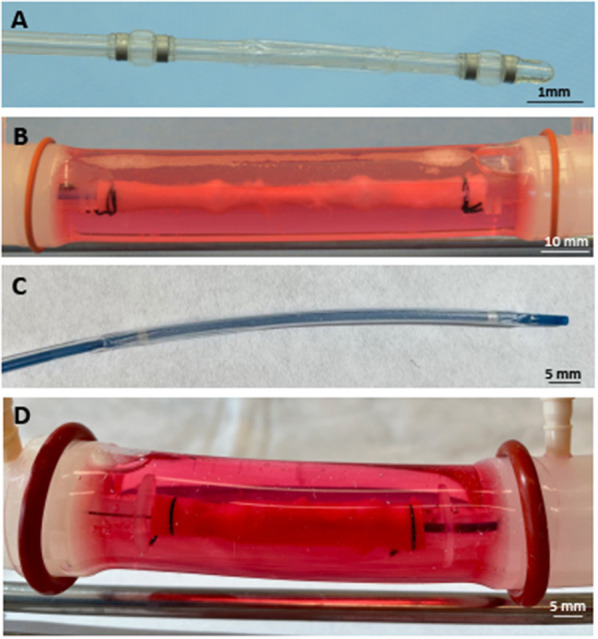
Deployed drug delivery devices. **(A)** Representative photograph of the perfusion catheter. **(B)** Image of the perfusion catheter system deployed within the explanted pig artery. Due to the optical clarity of the system, the proximal and distal occlusion balloons are clearly visible and can be identified in the artery. **(C)** Representative photograph of the drug-coated balloon. **(D)** Image of the drug-coated balloon deployed within the explanted pig artery.

To deliver paclitaxel locally to selected regions, a multi-lumen balloon perfusion catheter (Advanced Catheter Therapies, Chattanooga, TN) and a DCB (Lutonix, BD, Covington, GA) were utilized. The perfusion catheter, as described previously (33, 34), temporarily occludes the target area from blood flow by deploying occlusion balloons (Figure 2). The delivery of therapeutic agent with the perfusion catheter is accomplished by pressure differences from an increase in luminal pressure, which drives the drug molecules across tissue layers and into the medial wall. The inflation pressure of the DCB was determined by the manufacturers' specifications to achieve a 5–10% overstretch of the arterial lumen for 30 s of inflation. Prior to DCB deployment, the diameter of the explanted artery was evaluated by ultrasound. The position of the treated arterial segments was marked on the outer sheath of the vessel housing compartment to record the specific region of the harvested artery exposed to paclitaxel. *Ex vivo* treated arteries (*n* = 4 per time point) were harvested at 1 h post-treatment for pharmacokinetic evaluation.

### Pig Injury Model

This study was approved by the Institutional Animal Care and Use Committee (IACUC) and conformed to the current Guide for the Care and Use of Laboratory Animals. The experimental preparation of the animal model has been previously reported (35, 36). Four female pigs (12.3–14.1 kg) were anesthetized, and the right carotid artery was exposed under a sterile field. The caudal end of the right carotid artery was tied-off. Using micro scissors, a small incision was made to the right carotid artery and a 6 French (F) guide sheath was inserted. A NITREX™ 0.014 guidewire (ev3 Inc., Plymouth, MN) was inserted and, under fluoroscopic guidance, endothelial denudation using a 4 × 12 mm angioplasty balloon catheter (Abbot Vascular, Abbott Park, IL) was performed to the left and right iliac arteries. Following denudation, drug-delivery devices were tracked to each of the iliac arteries and deployed for 2 min. Antiplatelet therapy consisted of aspirin (40 mg/day) given orally 24 h before catheterization with continued dosing throughout the in-life phase of the study, while single-dose intra-arterial heparin (150 IU/kg) and lidocaine were administered at the time of catheterization. Animals were anesthetized and euthanized by intravenous Fatal-Plus (Vortech Ltd., Dearborn, MI) injection (85–150 mg/kg) at 1 h. Treated segments were excised following geographic landmarks determined by angiography and stored at −80°C.

### Quantification of Paclitaxel Tissue Concentrations

Paclitaxel tissue concentrations were quantified using a validated, previously described high-performance liquid chromatography (HPLC)-electrospray ionization- tandem mass spectrometry system assay (LC-MS/MS). The instrument setup and the assay principles have been described previously (37). The system was made up of a series 1260 HPLC system (Agilent Technologies, Santa Clara, CA) and an ABSciex 5000 triple-stage quadrupole mass spectrometer (ABSciex, Concord, ON). Paclitaxel-D_5_ was purchased from Toronto Research Chemicals (Toronto, ON) to be used as the internal standard. Untreated pig arteries were used to prepare calibration curves consisting of paclitaxel concentrations ranging from 0.5 to 100 ng/mL (38).

Briefly, 100 μL of sample was injected onto a 4.6 × 12.5 mm 5 μm extraction column (Eclipse XDB C-8, 5 μm particle size, Agilent Technologies, Palo Alto, CA). Samples were then washed with a mobile phase of 15% methanol and 85% 0.1% formic acid. The flow was 3 mL/min and the temperature for the extraction column was set to 65°C. After 1 min of washing the samples, the column switching valve (Rheodyne, Cotati, CA)was activated, and the analytes were then back-flushed from the extraction column onto a 150 × 4.6 mm C8, analytical column (Zorbax XDB C8, 3.5 μm particle size, Agilent Technologies, Palo Alto, CA). The following gradient was run: 60% methanol/0.01% formic acid to 98% methanol/0.01% formic acid within 2 min and stayed at 98% methanol/0.01% formic acid until 5.9 min. Hereafter, from 6.0 to 7.0 min, the analytical column was re-equilibrated to starting conditions. The flow rate was 1.050 mL/min and the analytical column was kept at 65°C. Paclitaxel was detected in the positive multi-reaction mode using the following ion transitions: m/z = 876.6 [M+Na]^+^ → 308.2. The internal standard, Paclitaxel-D_5_, was detected using the transition m/z = 881.6 [M+Na]^+^ → 313.1. Paclitaxel concentrations were quantified based on the analyte/ internal standard ratios using the calibration curves that were included in each batch. The LC-MS/MS instrument was controlled, ion chromatograms were recorded, and the analyte peaks were integrated using Sciex Analyst (version 1.6.2.). Calibration data were fit using a quadratic regression with 1/x weighting.

### Statistical Analysis

All values were expressed as mean ± standard deviation (SD). Quantitative data were analyzed with an unpaired *t*-test using GrapPad Prism 9 (GraphPad Software, La Jolla, CA, USA) and assuming the values follow a Gaussian distribution. A value of *p* ≤ 0.05 was considered statistically significant.

## Results

Figure 2 shows representative images of porcine carotid arteries being treated by a perfusion catheter and a DCB within the *ex vivo* bioreactor system. The explanted vessels were pulsed for 1 h in the *ex vivo* system, where they were subjected to physiological flow conditions. The flow consisted of a systolic pressure of 120 mmHg and a diastolic pressure of 80 mmHg (Figure 1B) with flow rates ranging from 15 to 150 ml/min (Figure 1C). The mean wall shear stress value ranged from 1.3 to 19.0 dynes/cm^2^, which is similar to reported pig wall shear stress values (39).

To evaluate tissue drug retention, treated arterial segments were removed 1 h post-drug delivery by the perfusion catheter and the DCB. Due to the optical clarity of the bioreactor, the treatment area could be clearly identified to ensure only the portion of the artery treated by the drug was excised (Figure 2). Arterial tissue paclitaxel concentrations of the *ex vivo* pig arteries were measured at 25.42 ± 9.74 ng/mg and 48.49 ± 24.09 for the perfusion catheter and the DCB, respectively.

**Figure 3:**
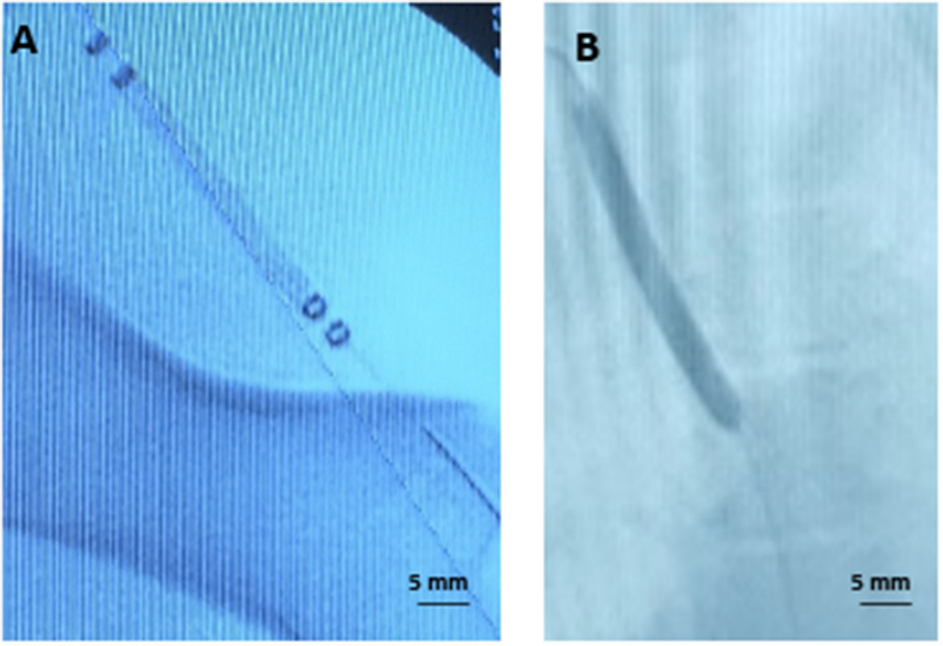
Representative angiographic images. **(A)** Angiogram of the perfusion catheter during delivery. **(B)** Angiogram of the drug-coated balloon during delivery.

**Figure 4:**
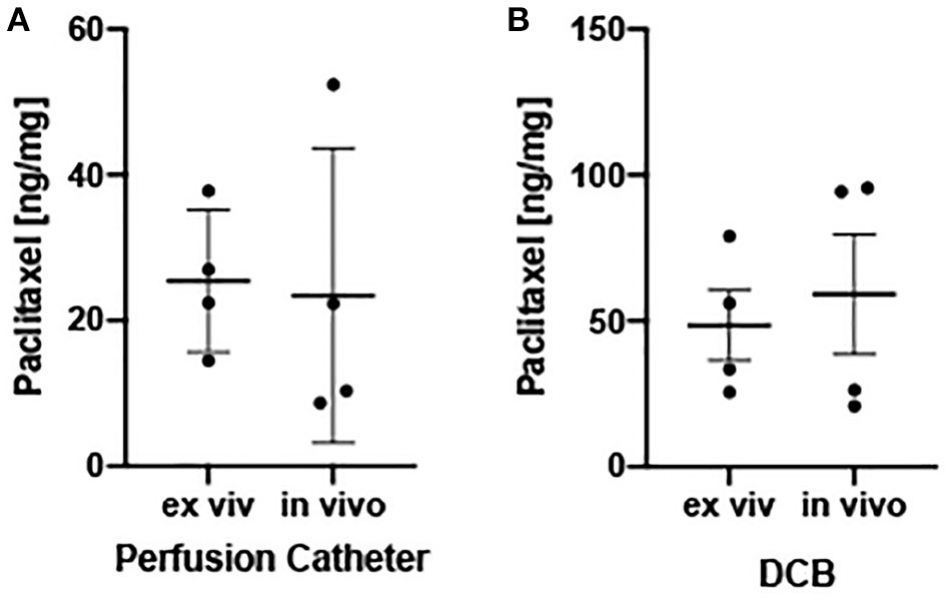
*Ex vivo* and *in vivo* arterial drug retention. **(A)** Scatter plots displaying measured arterial paclitaxel concentrations within the perfusion catheter-treated segments expressed as ng/mg. **(B)** Scatter plots displaying measured arterial paclitaxel concentrations within the drug-coated balloon-treated segments expressed as ng/mg. Each bar represents the mean ± standard deviation.

To validate tissue paclitaxel levels *ex vivo*, parallel *in vivo* studies were performed using the pig ilio-femoral injury model. Paclitaxel delivery using the perfusion catheter, with similar dosing and delivery parameters, and the DCB were repeated. An angiogram of the delivery devices within the ilio-femoral artery is shown in Figure 3. Arteries were again explanted at 1 h. *In vivo* arterial paclitaxel concentrations were measured at 23.43 ± 20.23 ng/mg for the perfusion-treated arteries and 59.23 ± 41.27 ng/mg for the DCB-treated arteries. Bar graphs displaying both the *ex vivo* and *in vivo* arterial paclitaxel concentrations are shown in Figure 4. Overall, no statistically significant differences were observed between paclitaxel measurements of arteries treated *ex vivo* vs. *in vivo* with either the perfusion catheter (*p* = 0.87) or the DCB (*p* = 0.67).

## Discussions

The study's main objective was to develop an *ex vivo* system capable of rapidly evaluating arterial drug levels comparable to their *in vivo* counterparts. The results demonstrate that paclitaxel can be delivered successfully to native porcine arteries within our bioreactor system using a DCB and a perfusion catheter. Similar arterial paclitaxel concentrations were observed between the *in vivo* and *ex vivo* models. Our arterial paclitaxel concentrations also compared to results reported in the literature (20, 40). Overall, these results demonstrate a viable platform for evaluating drug release kinetics of interventional vascular devices in an *ex vivo* setting.

This technology will serve as an intermediate step to rapidly assess the pharmacokinetics of drug delivery devices while limiting animal usage and cost of testing. For any short-term *in vivo* experiment (<7 days), months of preparation time is needed to obtain approval from the IACUC, order animals, quarantine, and schedule the procedure to implant and retrieve the device or tissue. Each step of this process (animal housing, staff support, operating table time) increases the cost and duration of the study. By using fresh swine arteries from local slaughterhouses, costs and animal usage are minimized to nearly zero. Fresh swine arteries are readily available in abundant quantities and typically at no cost.

The motivation for the current work is to demonstrate the viability of an *ex vivo* system for pharmacokinetic evaluation of acute arterial drug retention. The current paradigm for evaluating the pharmacokinetics of vascular devices (preclinical) was relatively non-existent prior to animal testing. Bench testing of non-stent devices (such as DCB and perfusion catheters) is confined to mechanical testing of balloons (burst pressure, fatigue), tracking of particulate matter, and biocompatibility of drugs and carriers using cell culture and static techniques (41–43). These factors are important in the development of new devices, but drug pharmacokinetics is the distinguishing factor of a successful device. The total drug-coated on a balloon can be quantified by it being eluted from the balloon into a saline bath of controlled volume. However, the transfer of the drug from the device to an artery differs greatly from those measurements. Simulated *in vitro* elution can be very inaccurate compared to *in vivo* elution. Many factors, such as blood-drug interaction, blood-tissue interaction, and drug-medium solvation, are not taken into account. These limitations are the reason drug delivery parameters are tested and verified *in vivo* by removing treated vessels at several time points to determine the acute transfer of drug and drug retention. Although these trials are essential in determining drug delivery, they are often prolonged, costly, and utilize many animals. New *ex vivo* methods to decrease the time and cost of evaluating such devices are desirable.

The developed *ex vivo* bioreactor system described here permits the use of explanted living native pig arteries as the test section. This study demonstrated that the explanted arteries can mimic the acute transfer of the drug from the device, which is the most significant aspect of acute non-stent platforms. The minimal therapeutic levels of tissue paclitaxel have been reported in the range between 0.01 and 1 ng/mg (26, 44). Although this range should be maintained for up to 28 days, the most influential aspect of pharmacokinetics is the acute transfer of paclitaxel from the device to the tissue since the majority of drug loss occurs in the first hours and days (45).

Non-stent delivery platforms are seen as an alternative approach to overcome the limitations of stents in the treatment of PAD. Stents in the periphery are subject to biomechanical stress increasing the risk of fracture and leading to device failure (12). Recently, DCBs have emerged as a therapeutic alternative for treatment of PAD (21). The primary advantage of this technology is the uniformity and short-term transfer of drug to the luminal surface without the need for a stent platform and a polymer carrier (18). The perfusion catheter also has similar characteristics in its ability to deliver the drug of choice uniformly, but also the added advantage of being able to load and deliver any drug at any concentration.

**Figure 5:**
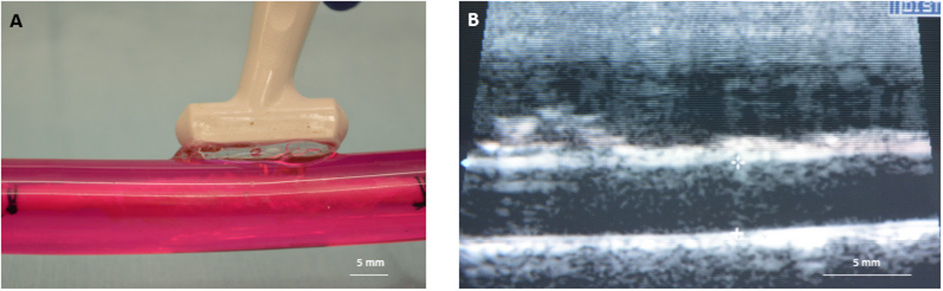
Diameter measurement of explanted arteries. **(A)** An ultrasound probe can be directly placed on the outer sleeve of the bioreactor housing to measure the diameter. **(B)** Ultrasound image showing the inner diameter of the explanted artery.

Other benefits of the system include: the ability to evaluate any commercially available vascular device, to run multiple experiments simultaneously, obtain results in days, and the low cost associated with performing *ex vivo* experiments as opposed to *in vivo* experiments. Additionally, due to the design of the system, the inner diameter of the explanted arteries can be monitored and measured using ultrasound (Figure 5). This feature is crucial for the deployment of DCBs as they are designed to be inflated with respect to the vessel diameter. Under deployment of the DCB can directly alter drug transfer to the artery.

**Figure 6:**
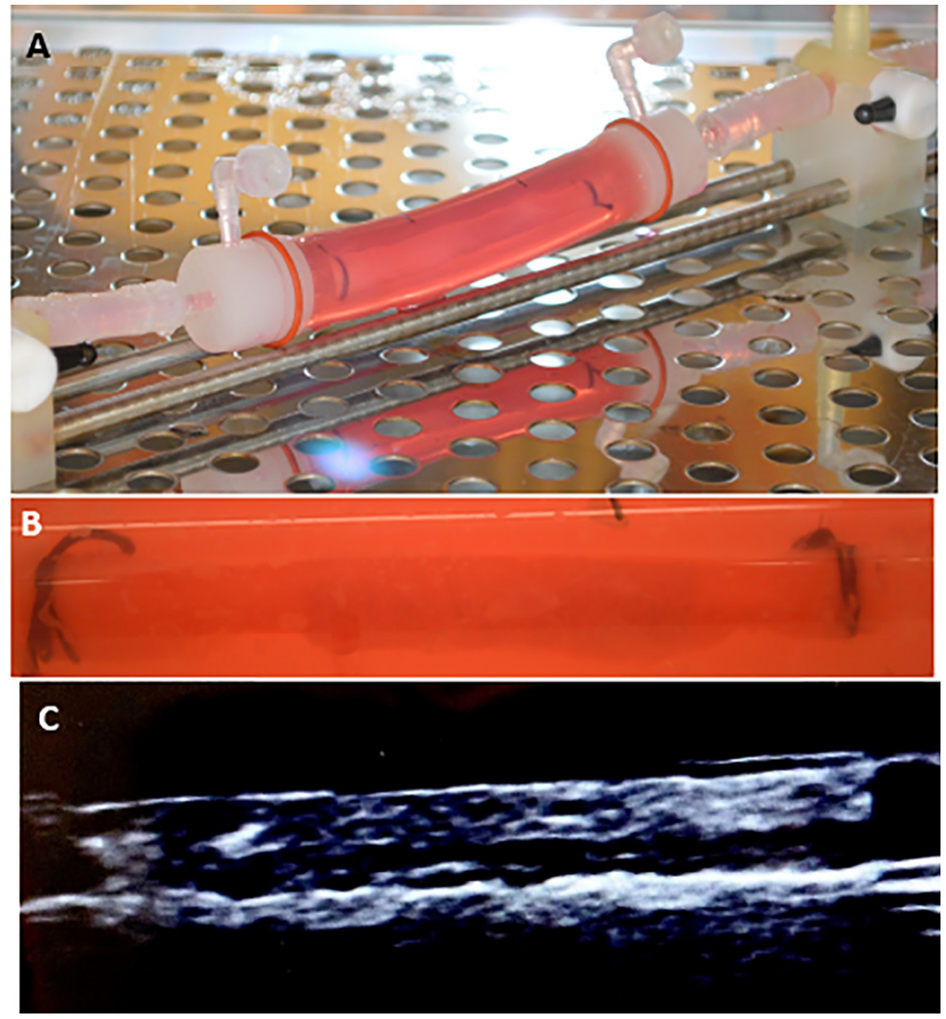
Arterial drug retention of a drug eluting stent. **(A,B)** Image of the drug eluting stent deployed within the explanted pig artery. **(C)** Ultrasound image showing the stented explanted artery.

Overall, our results demonstrated no significant differences between *ex vivo* and *in vivo* outcomes for the two tested drug delivery devices. To further highlight our *ex vivo* system, we performed additional studies on the drug retention of a commercially available DES (Synergy^TM^, Boston Scientific). The Synergy stents (*n* = 4) were successfully deployed in the *ex vivo* arteries, and arterial segments were analyzed for drug retention using pharmacokinetic analysis at 3-day time points (Figure 6). These results from the Synergy Monorail DES (everolimus): 1.862 ± 1.317 ng/mg, matched the manufacturers published results (46). These results further indicate the platform's ability to accurately measure arterial drug levels at extended time points while accommodating various devices, such as DES.

Some limitations of our system include that the working fluid is culture medium, rather than whole blood, and that the current studies only used healthy arteries (not diseased). Despite these limitations, evaluation of the drug delivery, elution, and retention can be accomplished. Off-the-shelf vascular devices can be deployed in an *ex vivo* native artery, conditioned at physiological mechanical conditions, and evaluated for drug pharmacokinetics.

To conclude, this system represents the first validated *ex vivo* pulsatile system to determine pharmacokinetics in a native blood vessel. We assert that this system can dramatically reduce the time and expense associated with *in vivo* testing of vascular devices, particularly in measuring and quantifying vessel drug retention. Future studies will include monitoring smooth muscle cell proliferation, endothelialization, and other biomarkers to understand the acute response of explanted arteries to arterial drug delivery systems. More advances in this type of technology will undoubtedly continue with the hope of reducing the preclinical trial expense and the time-to-market of many vascular devices.

## Data Availability Statement

The raw data supporting the conclusions of this article will be made available by the authors, without undue reservation.

## Ethics Statement

The animal study was reviewed and approved by University of South Alabama Institutional Animal Care and Use Committee.

## Author Contributions

SY, KC, CC, and EG contributed to the conception and design of the study. KC, CC, EG, and MA organized the database. KC, CC, UC, and SY performed the data analysis. SY and KC performed the statistical analysis and wrote the first draft of the manuscript. All authors contributed to the article and approved the submitted version.

## Conflict of Interest

SY serves on the Scientific Advisory Board of Advanced Catheter and has received grant support from Advanced Catheter Therapies, Lutonix, Inc, Alucent Biomedical, Toray Industries and Biosensors International. The remaining authors declare that the research was conducted in the absence of any commercial or financial relationships that could be construed as a potential conflict of interest.
